# Machine learning estimation of crude oil viscosity as function of API, temperature, and oil composition: Model optimization and design space

**DOI:** 10.1371/journal.pone.0282084

**Published:** 2023-02-17

**Authors:** Daihong Li, Xiaoyu Zhang, Qian Kang

**Affiliations:** 1 School of Civil and Environmental Engineering, University of New South Wales, Sydney, NSW, Australia; 2 China Gezhouba Group Three Gorges Construction Engineering Co., Ltd., Yichang, China; Wuhan University of Technology, CHINA

## Abstract

Measurement of viscosity of crude oil is critical for reservoir simulators. Computational modeling is a useful tool for correlation of crude oil viscosity to reservoir conditions such as pressure, temperature, and fluid compositions. In this work, multiple distinct models are applied to the available dataset to predict heavy-oil viscosity as function of a variety of process parameters and oil properties. The computational techniques utilized in this work are Decision Tree (DT), MLP, and GRNN which were utilized in estimation of heavy crude oil samples collected from middle eastern oil fields. For the estimation of viscosity, the firefly algorithm (FA) was employed to optimize the hyper-parameters of the machine learning models. The RMSE error rates for the final models of DT, MLP, and GRNN are 40.52, 25.08, and 30.83, respectively. Also, the R^2^-scores are 0.921, 0. 978, and 0.933, respectively. Based on this and other criteria, MLP is chosen as the best model for this study in estimating the values of crude oil viscosity.

## 1. Introduction

For simulation of oil flow in different media such as in the well, pipeline, and processing, the viscosity of oil plays crucial role, and the accuracy of simulations depends on the accuracy of viscosity determination. Indeed, robust, and reliable models are required to estimate the viscosity of oils (e.g., crude oils) for a wide range of oil sources. Sometimes, the gas might be dissolved in the oil, and its viscosity estimation would be challenging. Khemka et al. [1] proposed a method for viscosity modeling of light crude oils containing dissolved gas. They used one-parameter friction theory for estimation of viscosity of oil under gas injection. The accuracy of the viscosity prediction is of great importance, and unrealistic models would make pitfalls in simulation of oil behavior such as those in the reservoir.

Considering the composition of the crude oils which contain a range of hydrocarbons, the realistic models should be able to take into account the composition of the crude oil when estimating the viscosity values [2]. Kamel et al. [2] developed a methodology for estimation of heavy crude oils based on compositional models. Their model was of empirical nature and outperformed other oil estimators such as corresponding-state, *EoS*, etc. The statistical analysis of their model revealed that the average relative error of the fitting was 3.8%. Development of holistic models for estimation of viscosity of oils from different sources is demanding, and advanced methods such as machine learning (ML) could be employed for this application. There are limited developed ML techniques for prediction of crude oils properties based on compositional data, and there is a research gap in this area to be addressed.

Analytics are losing ground to machine learning (ML) techniques in the scientific community owing to the power of ML methods in data analytics applications [3]. Different versions of artificial neural networks (NN), models based on decision trees, and other linear and non-linear models are all examples of these methods in action. Now, machine learning models may look into any issue with a set of inputs and a set of desired outcomes [4]. Using a wide variety of methods, these models determine if there is a connection between variables [5–7]. In this research, three methods are implemented: Multiple Layers Perceptrons (MLP), Decision Tree (DT), and GRNN for estimation of crude oil viscosity based on compositional data [8].

The term "multilayer perceptron" (MLP) describes a specific type of neural network that consists of several layers of perceptrons. Multi-layer Perceptrons (MLPs) are an artificial neural network type that feeds new information into the network as it is MLP consists of at least three layers of inputs, outputs, and hidden layers. Nodes in the input layer are not switched on; rather, they stand in for the actual data point. A d-dimensional vector reflecting the data point would result in a d-dimensional number of nodes in the input layer [9, 10].

Radial basis function neural networks are utilized in the GRNN, which is yet another model that is based on neural networks (RBF). RBF uses a probabilistic framework to simulate the dependent variables of a regression function. It is impossible for other neural networks to reach a local optimum due to its probabilistic construction [11].

A decision tree (DT) is a ML technique used to solve classification and regression roots. The decision tree has advantages over other classification systems since it uses a hierarchical decision-making framework rather than merely grouping features (or bands) together. To solve many kinds of machine learning issues, the DT provides a hierarchical and understandable paradigm. We start at the root of the DT and move our way down the tree based on the value of each characteristic in each node’s subtree. This procedure is repeated until no more leaves or nodes remain [8, 12–14].

The main objective of the current study is to develop a machine learning strategy for estimation of crude oil viscosity based on compositional data. For the first time, the machine learning methods of Multiple Layers Perceptrons (MLP), Decision Tree (DT), and GRNN are used and tuned to estimate the viscosity as the target parameter, while the input parameters are the oil compositions and its physical properties. Statistical analysis is then performed to evaluate the performance of the tuned machine learning models, and select the best one.

## 2. Data set of crude oil

We used a dataset derived from research described in [2] describing the measurements and correlations of heavy crude oils viscosity versus a number of input parameters. A subset of training data is divided into a subset of testing data. An analysis of 28 samples of heavy crude oil supplied from the Middle East is included in the collection. As part of the construction of the model, 196 separate measurements of viscosity were collected at temperatures ranging from 20 to 80 degrees Celsius. A total of 47 additional viscosity measurements were performed to validate the model. Additionally, previously reported methods for estimating the viscosity of heavy oils were validated based on the composition and viscosity results. Heavy-oil viscosities were forecast using these methods. The dataset used in this work are listed in Tables 1 and 2.

**Table 1:** The training dataset used in this work [2].

No.	API at 15°C	%C_4_	%C_5_	%C_6_	%C_7+_	MW_C7+_	SG_C7+_	ρ (g/cc) at 20°C	μ (cP) at 20°C	μ (cP) at 30°C	μ (cP) at 40°C	μ (cP) at 50°C	μ (cP) at 60°C	μ (cP) at 70°C	μ (cP) at 80°C
1	20.4	1.02	2.57	3.37	93.04	326.40	0.94	0.930	200.59	106.07	62.6	40.177	27.268	19.367	14.258
2	20.0	0.04	0.44	1.62	97.90	331.22	0.93	0.933	243.11	131.9	76.875	48.461	32.351	22.637	16.5
3	19.6	0.25	1.57	3.94	94.24	343.10	0.94	0.935	322.46	171.13	96.462	58.975	38.663	26.759	19.225
4	19.5	0.00	0.12	1.16	98.72	295.70	0.94	0.935	312.40	158.25	88.722	54.503	35.498	24.334	17.456
5	18.7	0.08	1.13	2.09	96.70	342.65	0.94	0.941	232.12	126.17	74.171	46.59	30.978	21.568	15.598
6	18.6	0.35	1.62	3.21	94.81	338.41	0.95	0.941	196.73	108.36	64.539	41.088	27.617	19.408	14.192
7	18.0	0.46	1.23	2.06	96.25	353.31	0.95	0.945	319.57	262.18	140.28	81.78	51.562	34.484	24.369
8	18.0	0.35	1.14	1.95	96.56	353.44	0.95	0.945	285.66	151.65	87.573	54.331	35.767	24.71	17.801
9	18.0	1.05	1.53	1.92	95.50	347.26	0.95	0.945	396.52	171.08	97.209	59.809	39.16	26.93	19.336
10	17.9	0.15	1.65	3.72	94.49	357.24	0.95	0.945	516.01	205.44	115.72	70.104	45.172	30.612	21.64
11	17.6	0.18	1.16	2.73	95.93	343.69	0.95	0.947	309.50	162.45	92.869	56.966	37.234	25.471	18.215
12	17.4	0.46	0.49	1.95	97.10	367.35	0.95	0.949	494.24	249.43	137.83	81.972	52.014	34.807	23.512
13	17.1	0.00	0.23	1.19	98.58	345.72	0.95	0.951	891.90	431.67	209.77	118.36	72.911	47.536	32.358
14	16.8	0.17	0.73	2.23	96.87	354.55	0.96	0.952	374.80	192.84	108.31	65.532	42.155	28.601	20.13
15	16.7	0.16	0.96	1.87	97.00	351.13	0.96	0.953	382.14	197.41	110.22	66.462	42.623	28.759	20.242
16	16.4	0.09	0.43	1.21	98.27	372.04	0.96	0.955	521.79	259.03	141.78	83.945	53.008	35.422	24.717
17	16.2	0.00	0.09	0.40	99.51	380.46	0.96	0.956	1320.2	607.26	291.99	159.21	95.079	60.171	40.503
18	16.0	0.09	1.25	1.42	97.23	378.29	0.96	0.958	552.91	272.47	147.95	86.964	54.608	36.261	25.209
19	15.9	0.09	0.34	1.04	98.54	367.17	0.96	0.958	535.47	264.89	143.88	85.346	53.702	35.705	24.913
20	15.8	0.18	0.68	2.00	97.14	335.27	0.96	0.959	541.02	266.69	144.7	84.987	53.336	35.294	24.511
21	14.9	0.15	0.32	1.37	98.16	369.29	0.97	0.965	788.58	375.15	196.19	111.31	68.012	43.975	29.95
22	14.9	0.09	0.39	1.21	98.31	377.01	0.97	0.965	767.13	363.2	191.05	108.93	66.528	46.093	29.308
23	14.8	1.01	1.95	2.62	94.42	412.79	0.97	0.966	1294.6	420.23	217.33	123.11	74.758	48.121	32.683
24	14.8	0.69	1.32	1.91	96.09	361.14	0.97	0.965	895.64	591.89	299.11	165.25	98.236	61.867	41.218
25	14.8	0.11	0.21	0.96	98.72	364.62	0.97	0.966	746.11	353.31	185.22	105.68	64.809	42.081	28.698
26	14.5	0.13	0.25	1.05	98.58	362.00	0.97	0.968	1022.4	430.23	221.74	124.31	74.883	47.925	32.261
27	14.3	0.21	0.18	1.45	98.16	355.03	0.97	0.969	930.79	466.73	238.63	132.32	79.264	50.546	33.914
28	13.4	0.05	0.05	0.39	99.52	384.46	0.98	0.975	1430.6	632.54	312.66	170.03	99.442	62.18	41.074

**Table 2:** The whole test dataset [2].

No.	API at 15°C	%C_4_	%C_5_	%C_6_	%C_7+_	MW_C7+_	SG_C7+_	ρ (g/cc) at 20°C	μ (cP) at 20°C	μ (cP) at 30°C	μ (cP) at 40°C	μ (cP) at 50°C	μ (cP) at 60°C	μ (cP) at 70°C	μ (cP) at 80°C
1	19.58	0.006	0.028	0.034	0.932	379.385	0.942	0.933	274.58	146.84	85.538	53.158	34.972	24.021	
2	19.53	0.009	0.036	0.040	0.915	368.718	0.938	0.928	208.19	114.65	68.41	43.491	29.153		
3	19.34	0.010	0.042	0.042	0.906	382.840	0.947	0.936	271.07	145.17	84.792	52.823	35.038		
4	18.67	0.007	0.034	0.032	0.927	374.896	0.948	0.939	379.76	196.12	110.99	67.072	42.829		
5	18.57	0.012	0.032	0.033	0.923	385.901	0.948	0.939	372.14	193.18	109.03	62.307			
6	17.75	0.043	0.036	0.038	0.884	418.224	0.958	0.946	524.35	263.96	145.73	86.411	55.436		
7	17.63	0.010	0.037	0.032	0.921	391.993	0.956	0.945	420.58	215.94	121.42	73.182	47.545		
8	17.56	0.009	0.039	0.040	0.912	400.445	0.957	0.947	567.13	282.52	154.97	91.285	57.671		
9	16.06	0.001	0.018	0.029	0.952	365.678	0.963	0.957	523.01	256.58	139.61	81.975	51.541	34.149	23.588

## 3. Methods of computations

**Fig 1:**
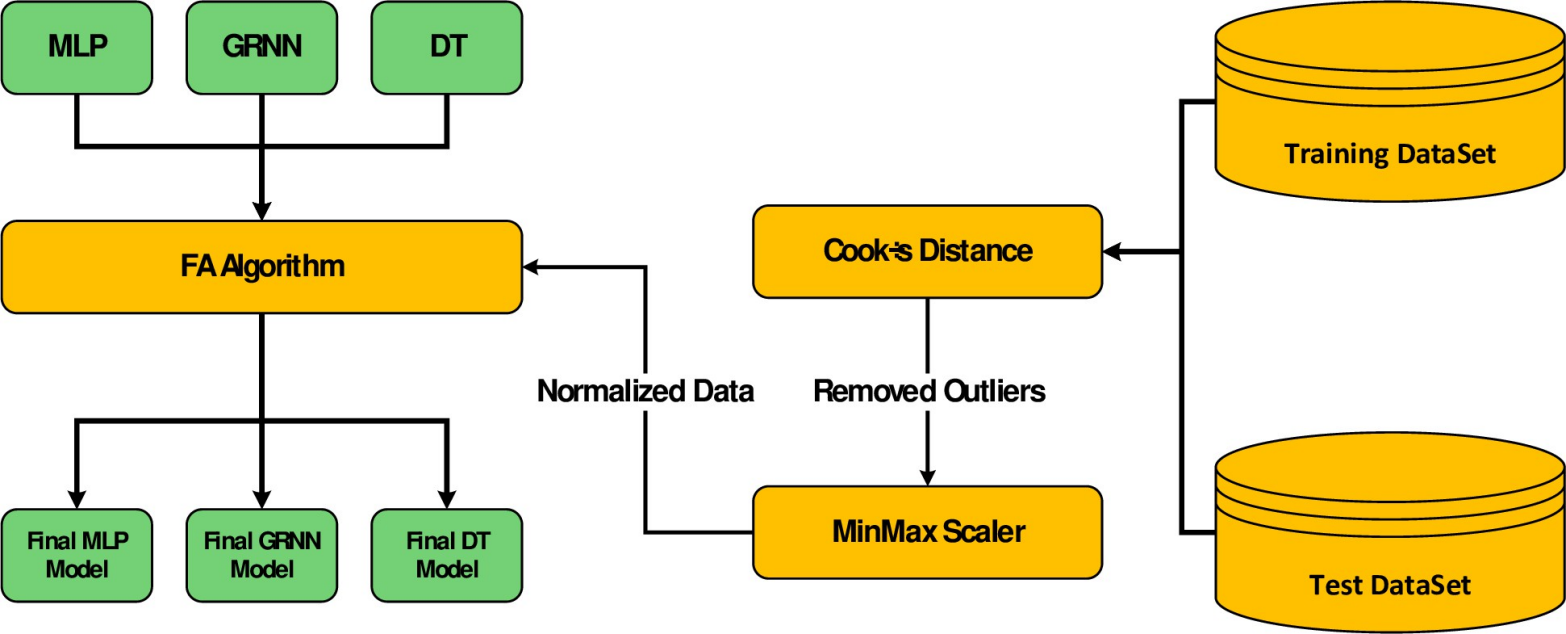
Methodology framework for correlation of crude oil viscosity.

In this research, the data are first pre-processed with the help of Cook’s distance and Min-Max normalization methods, and the prepared data sets are tested with the models in order to obtain optimal configurations. For this, we use the metaheuristic FA (firefly algorithm) method. In the following section, the building blocks of this innovative architecture shown in Fig 1 are introduced in detail.

### 3.1. Decision tree

**Fig 2:**
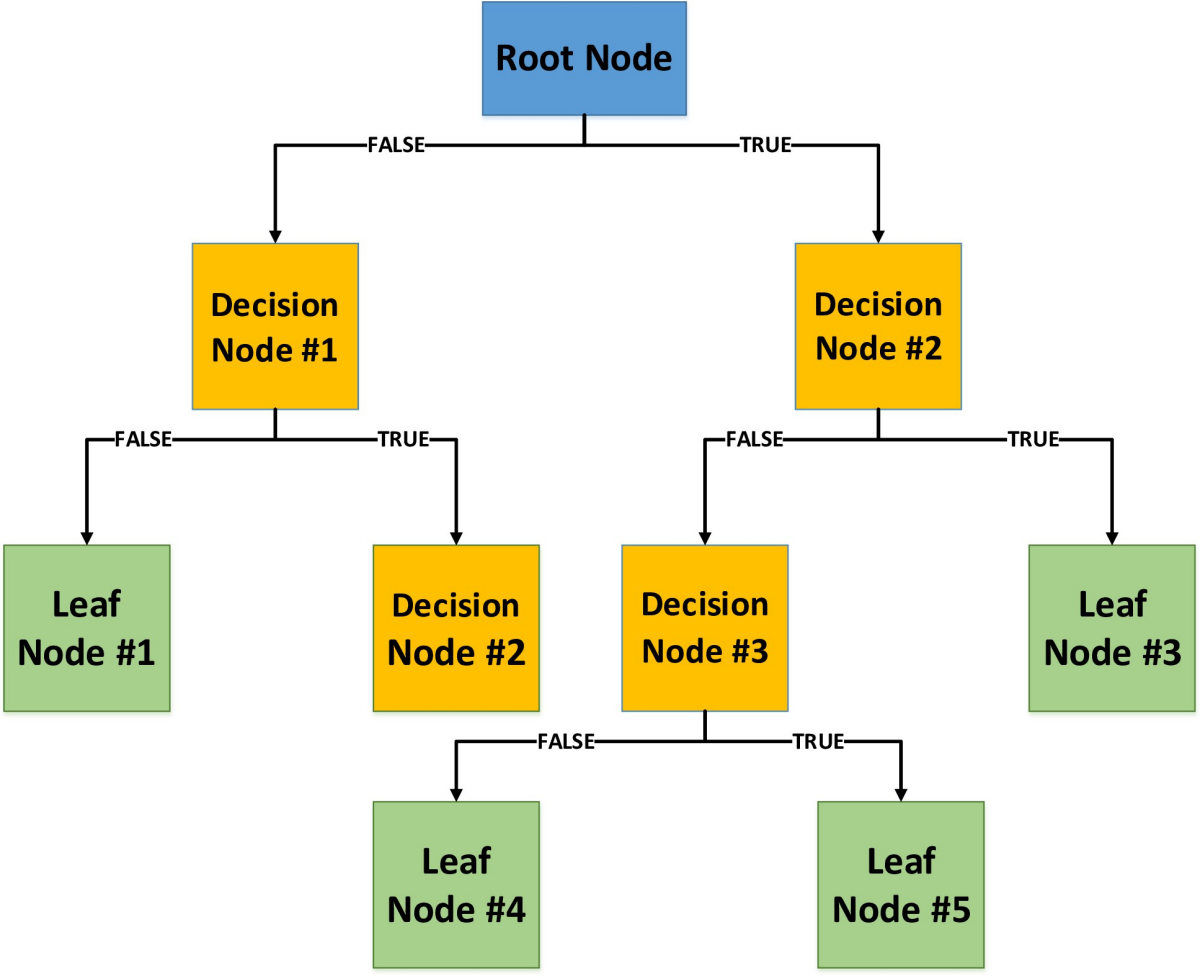
Structure of decision tree model.

DT is a widely accepted learning technique that can address a variety of problems. A DT is made up of three parts: a root (start) node, several internal (decision) nodes, and several leaf (terminal) nodes. The model’s output is represented by the leaf (terminal) nodes, while new information is introduced into the network at its root node [15]. There are some "decision nodes" in between the "root" and "leaf" nodes. In a typical network, information starts at the root node and travels outwards through the intermediate nodes until reaching the final node. The algorithm receives data as input and proceeds to construct a tree by a process of splitting, pruning, and terminating branches [12–14, 16, 17]. These actions start at the root node and progress till a certain condition has been achieved in the process. Fig 2 depicts a simplified decision tree conceptually.

### 3.2. Multilayer perceptron and GRNN

The concept of artificial neural networks was conceived in 1943 [18]. The perceptron, the first functional artificial neural network, was unveiled in 1958 [19]. The use of neural networks has increased in prominence since 1986 [20]. Neural networks use neurons as their fundamental building element since they are modelled after the nervous system. A variety of neural networks can be formed based on the connections, neuron model, and weight modification methods [10, 21]. Methods such as the Multilayer Perceptron (MLP) of artificial neural networks (ANN) could be employed to mimic the possible hidden correlations between the in and out data of processes [8, 10, 22].

Updates and optimizations based on work complexity enable a variable approach to hidden layer size. The MLP system’s artificial neurons are structured in a three-layered network [8, 10, 23].

The following equation is used to determine neuron input weights [22]:

$$z=x_{1}w_{1}+\cdots +x_{n}w_{n}=X^{T}W$$

The activation function, *f(z)*, can be calculated using a number of continuously differentiable functions, including the more modern *ReLU*, which is widely employed in the method of deep learning [8, 24, 25].

The GRNN model is a type of NN based on the radial basis function (RBF). RBF models the dependent variables in a regression problem using a probabilistic framework. Because of their probabilistic design, other neural networks are vulnerable to local optimum [26].

### 3.3. Firefly algorithm (FA) optimization approach

The firefly optimization algorithm (FA) is an innovative meta-heuristic algorithm that takes its name and inspiration from the flashing light of a firefly. The algorithm has many similarities to other swarm intelligence approaches like PSO, BFO, and others, but is easier to understand and implement. Accurately, FA simultaneously discovers both global and local optimums. Yang et al. developed and published this algorithm in [27–29]. Its primary advantage is that it is based on global communication among swarming particles (i.e., fireflies), making it appear more successful in multi-objective optimization. Yang et al. [29] go over the theoretical and technical aspects of the proposed method in greater detail [30].

## 4. Results and discussion

**Fig 3:**
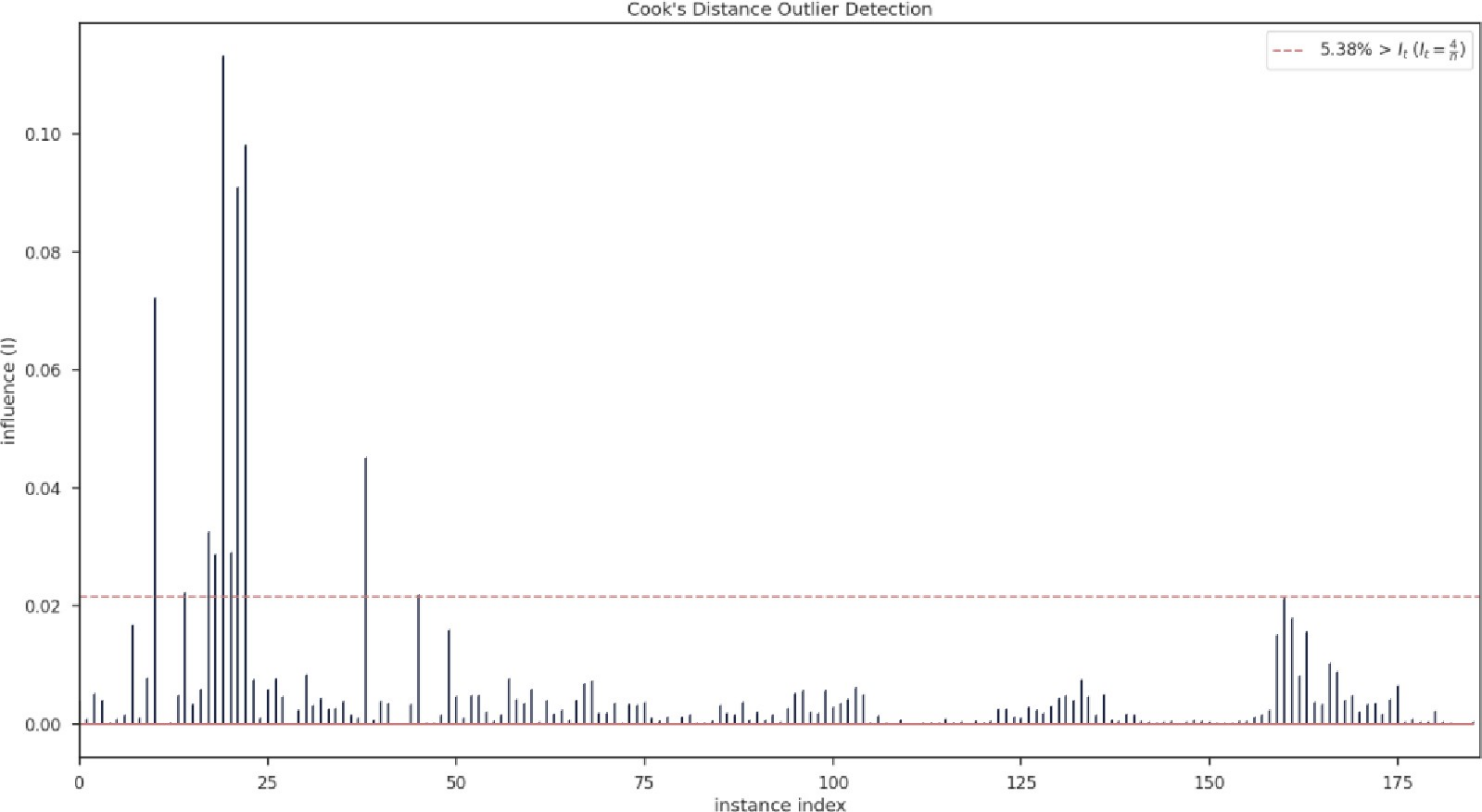
Demonstration of Cook’s distance outlier detection.

As mentioned in the explanation of the proposed method, before normalization, the existing data are evaluated with the help of Cook’s distance in the field of outliers, the result of which is shown in Fig 3. This figure shows that only 5% of the data as outliers should be removed from the data set in order to get better results of the modeling.

After the pre-processing of the dataset, with the help FA algorithm that was explained earlier, the models are optimized and tuned with their hyper-parameters to obtain the final models, the results of these models in terms of R-squared emissivity and error rates in the Tables 3 and 4 are displayed. It is seen that the MLP model has better accuracy in estimation of oil viscosity compared to DT and GRNN models. The statistical parameters including R^2^, MAE, RMSE, and MAPE confirm the accuracy of the tuned MLP model for this particular application in petroleum engineering.

**Table 3:** The comparisons of three developed models.

Models	MAE	RMSE	MAPE
*DT*	28.68	40.52	0.241
*GRNN*	20.41	30.83	0.177
*MLP*	19.06	25.08	0.156

**Table 4:** Final values of R^2^-scores for three models.

Models	Test score	Train score
*DT*	0.921	0.909
*GRNN*	0.933	0.903
*MLP*	0.978	0.912

**Fig 4:**
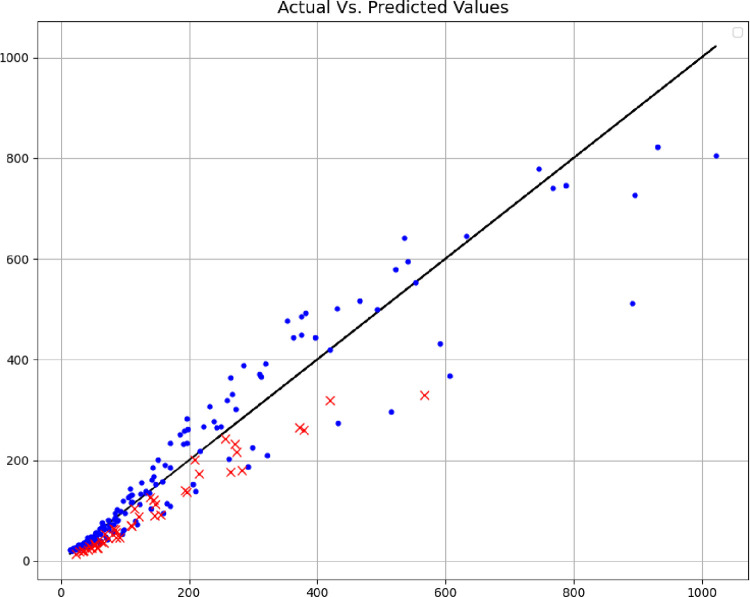
Actual Vs. estimated outputs (DT model).

**Fig 5:**
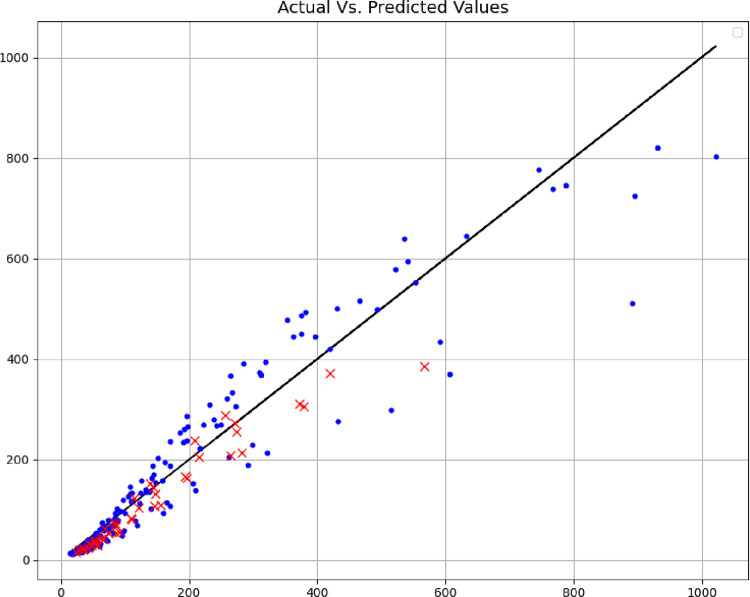
Actual Vs. estimated outputs (GRNN model).

**Fig 6:**
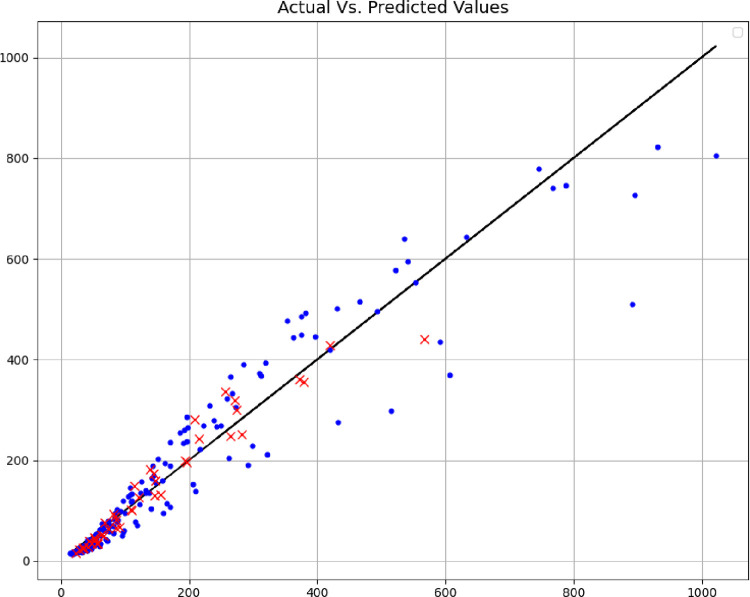
Actual Vs. estimated outputs (MLP model).

**Fig 7:**
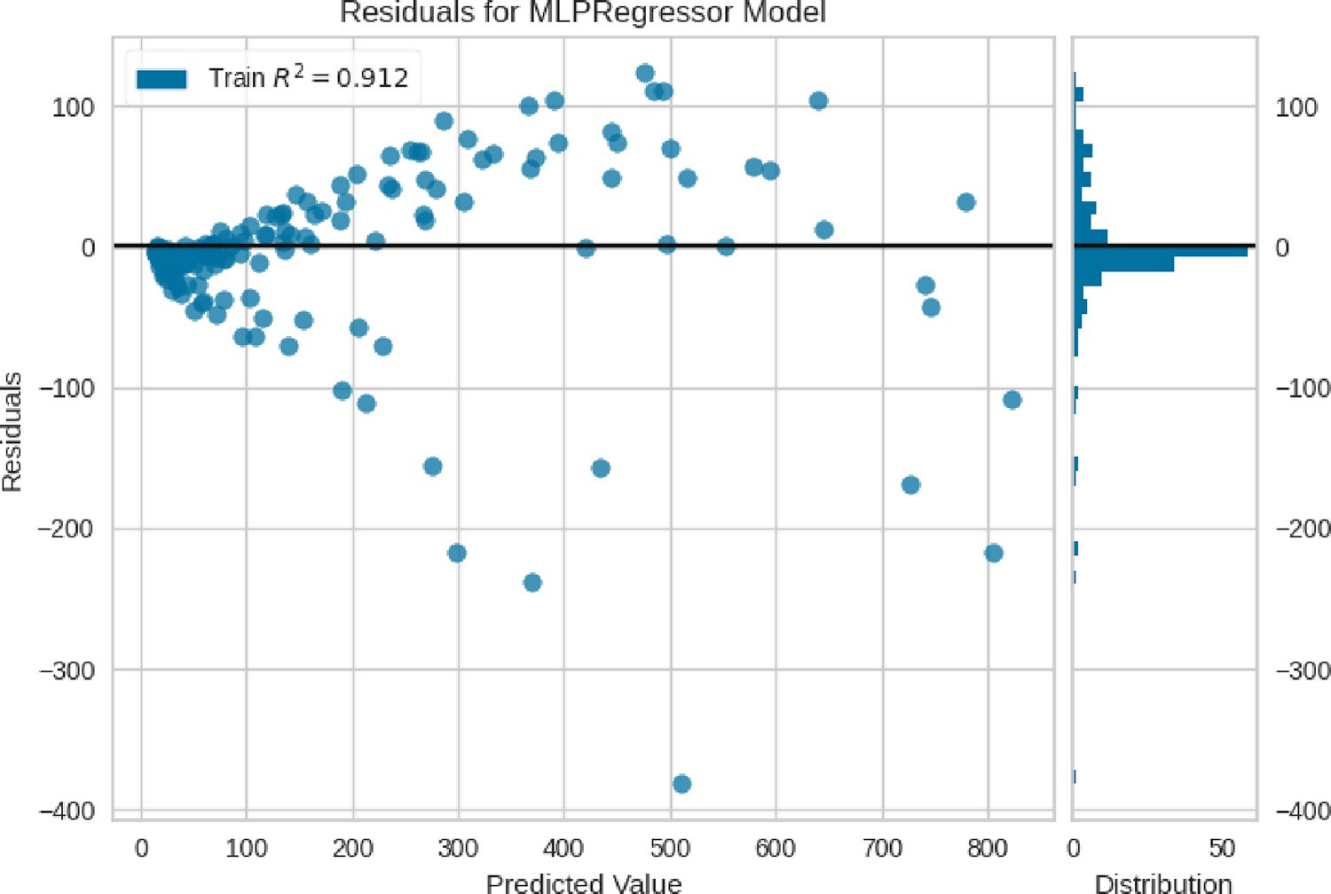
Residual plot of tuned MLP model.

In addition, the comparisons of the expected values and the predicted values are shown in Figs 4–6, where the blue points are the training data, and the red points are the test data. The comparison of these methods shows the fact that the models are very close to each other in terms of training data, but with accuracy in the test data, the MLP model can be considered the best model, therefore, the rest of the analyses are done with this model. Among other things, the residuals of this final model are shown in Fig 7.

**Fig 8:**
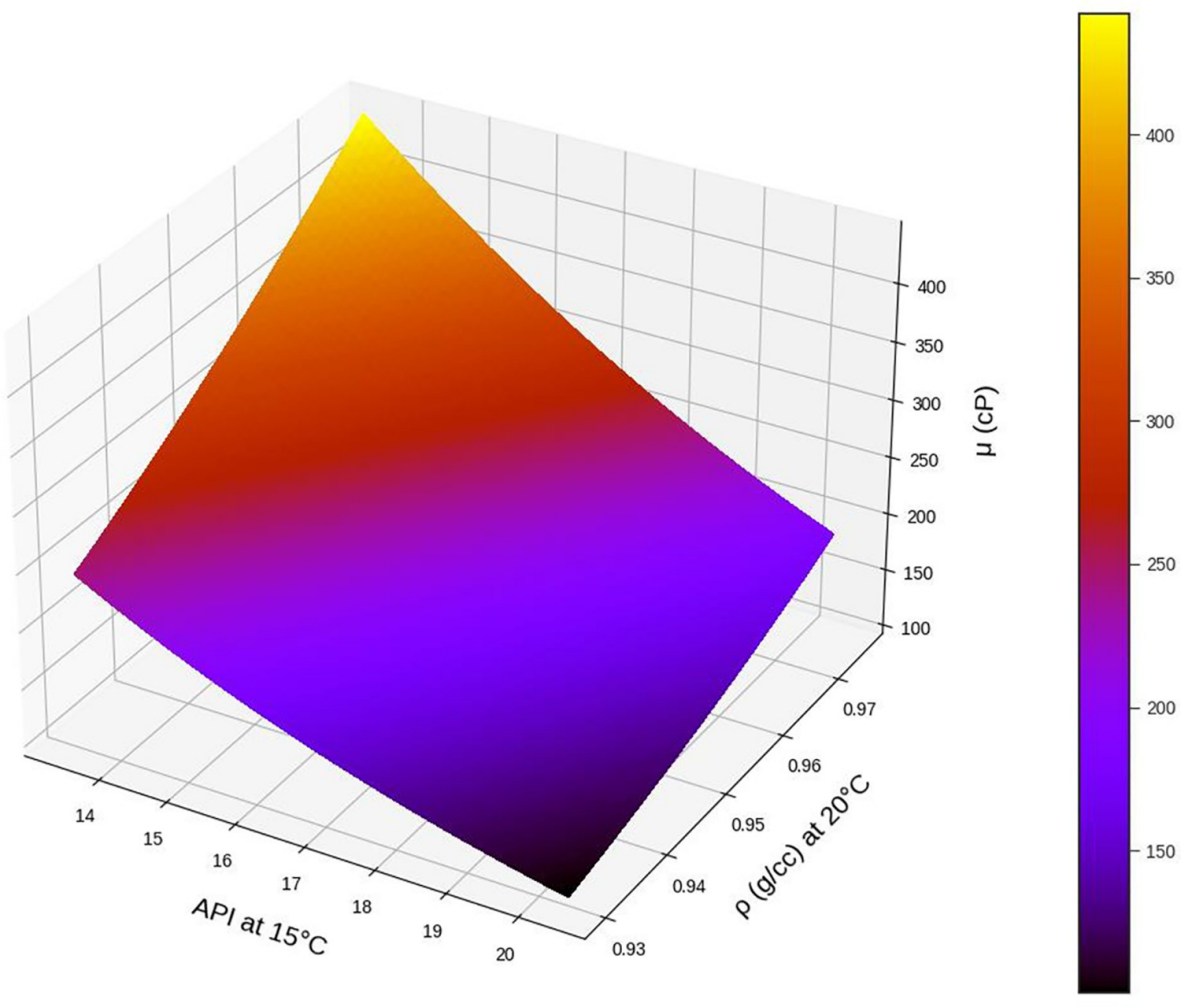
Effect of API and ρ (g/cc) on the output. %C4 = 0.35, %C5 = 1.62, %C6 = 3.21, %C7+ = 94.81, MWC7+ = 338.41, SGC7+ = 0.95, T (°C) = 30.

**Fig 9:**
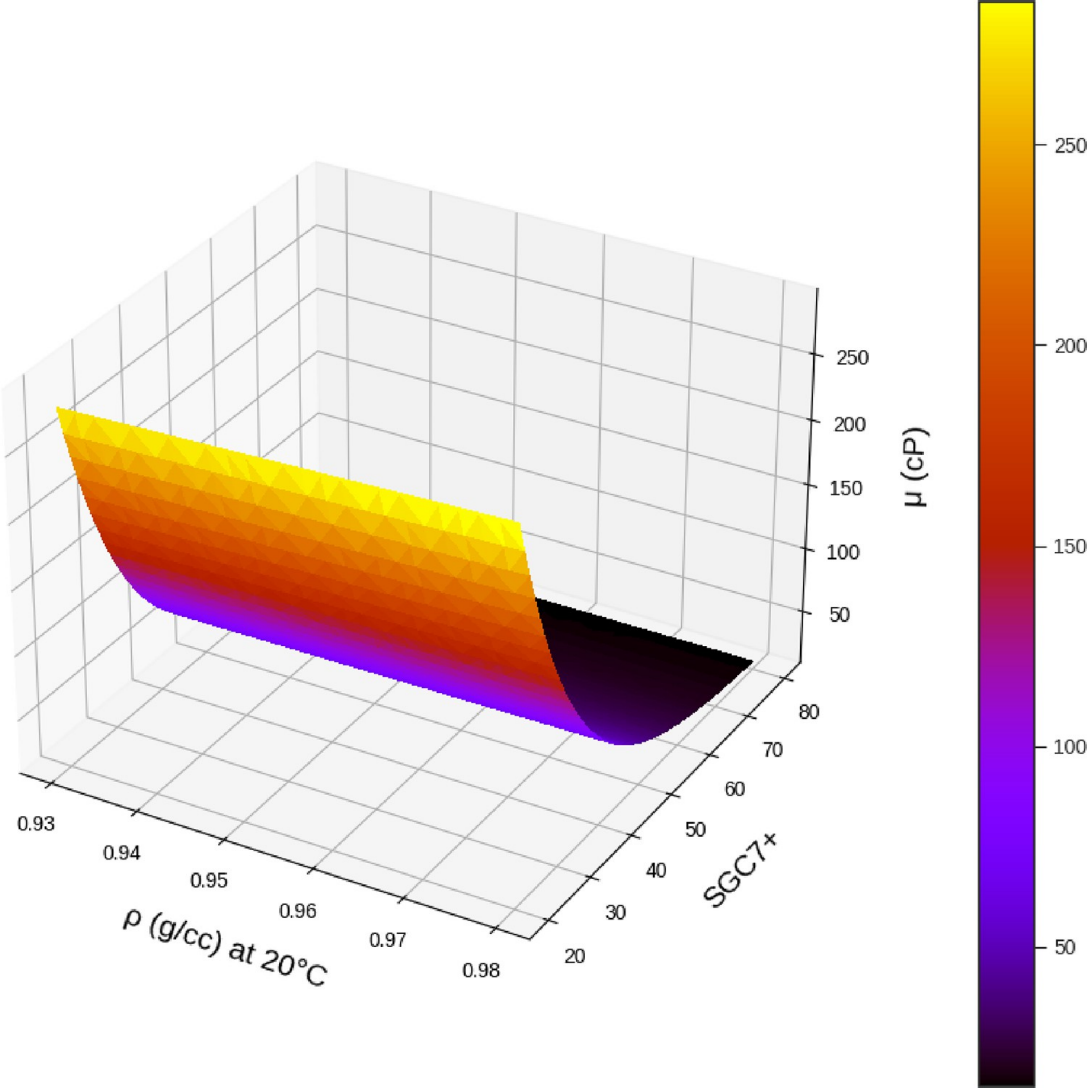
Influence of SGC7+ and ρ (g/cc) on the output. API = 18.6, %C4 = 0.35, %C5 = 1.62, %C6 = 3.21, %C7+ = 94.81, MWC7+ = 338.41, T (°C) = 30.

**Fig 10:**
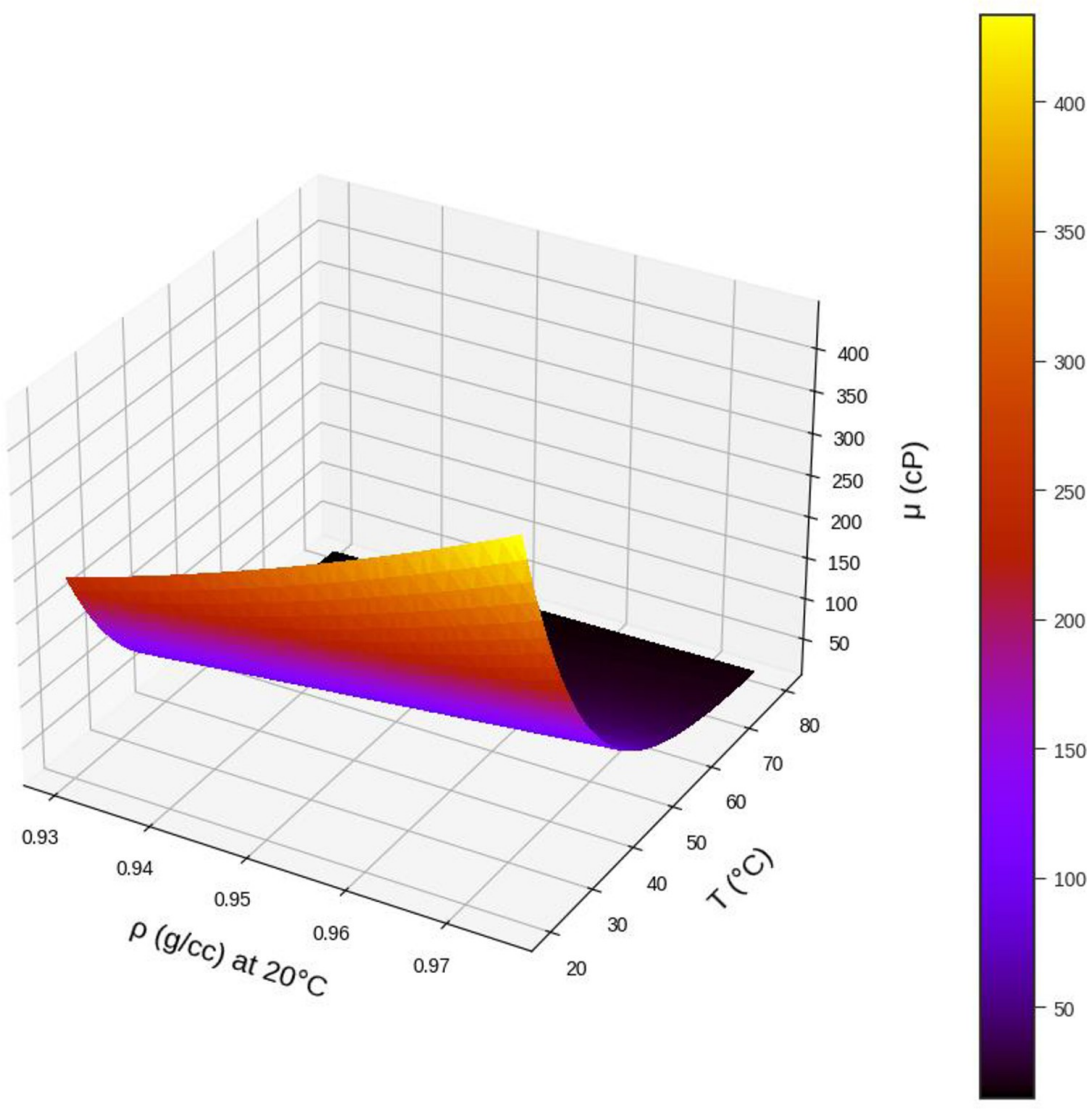
Effect of temperature and ρ (g/cc) on the output. API = 18.6, %C4 = 0.35, %C5 = 1.62, %C6 = 3.21, %C7+ = 94.81, MWC7+ = 338.41, SGC7+ = 0.95.

**Fig 11:**
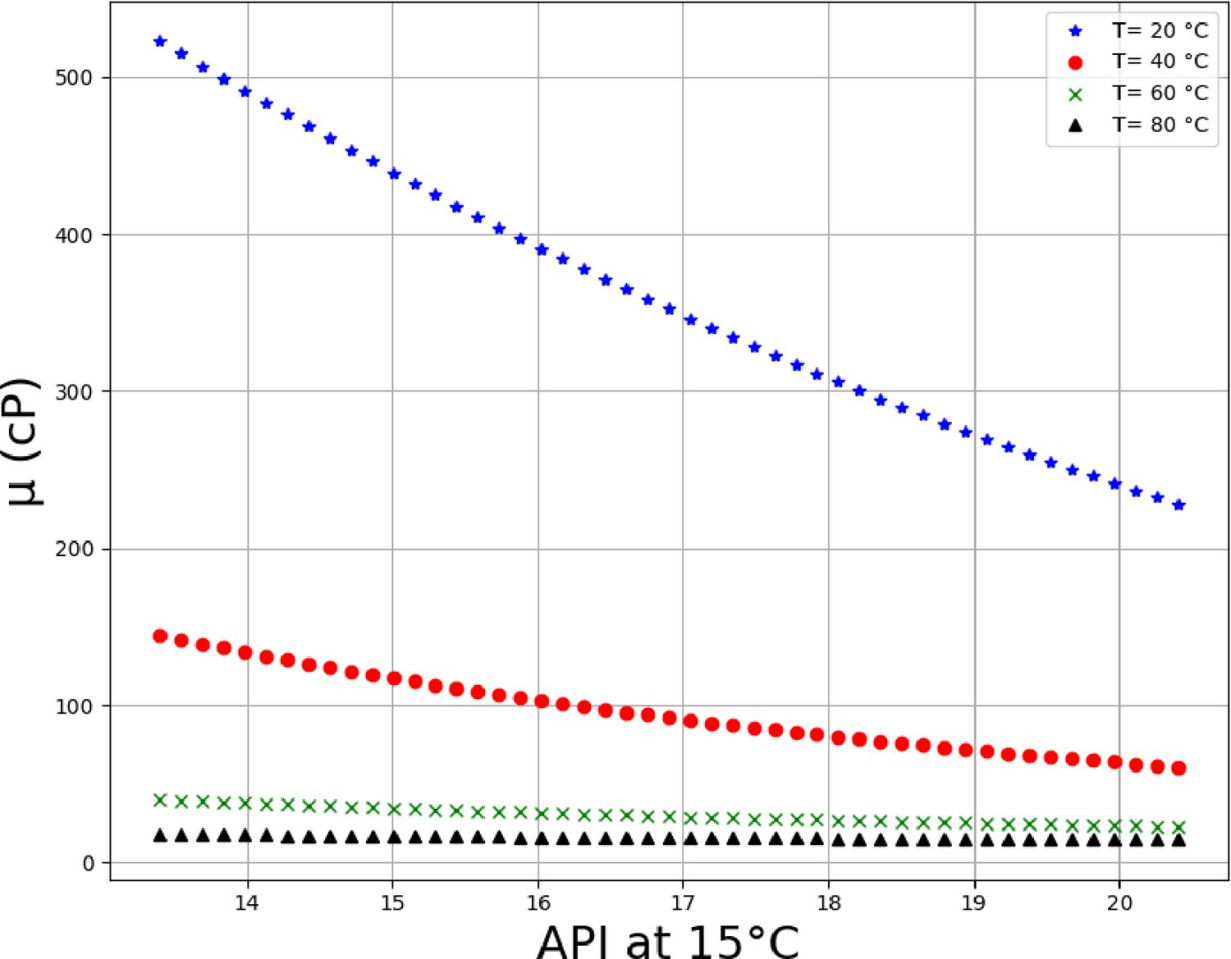
Trends of API on multiple temperature levels (%C4 = 0.35, %C5 = 1.62, %C6 = 3.21, %C7+ = 94.81, MWC7+ = 338.41, SGC7+ = 0.95, ρ (g/cc) at 20°C = 0.941).

**Fig 12:**
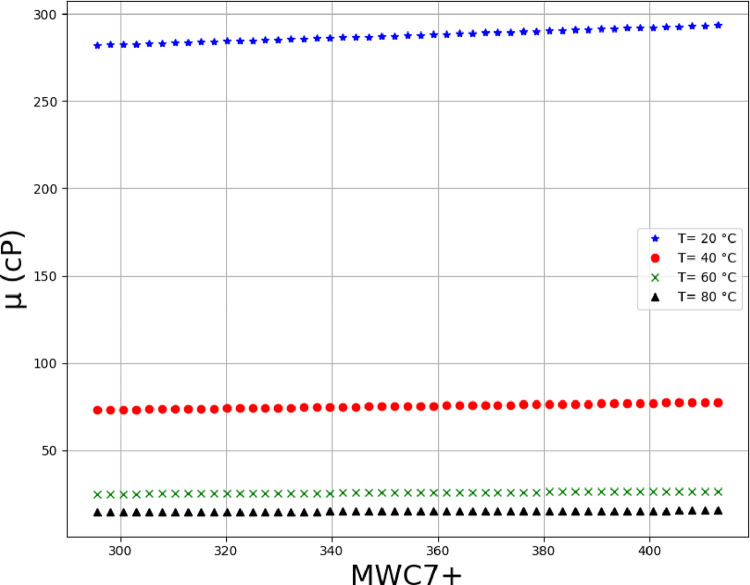
Trends of MWC7+on multiple temperature levels (API = 18.6, %C4 = 0.35, %C5 = 1.62, %C6 = 3.21, %C7+ = 94.81, SGC7+ = 0.95, ρ (g/cc) at 20°C = 0.941).

**Fig 13:**
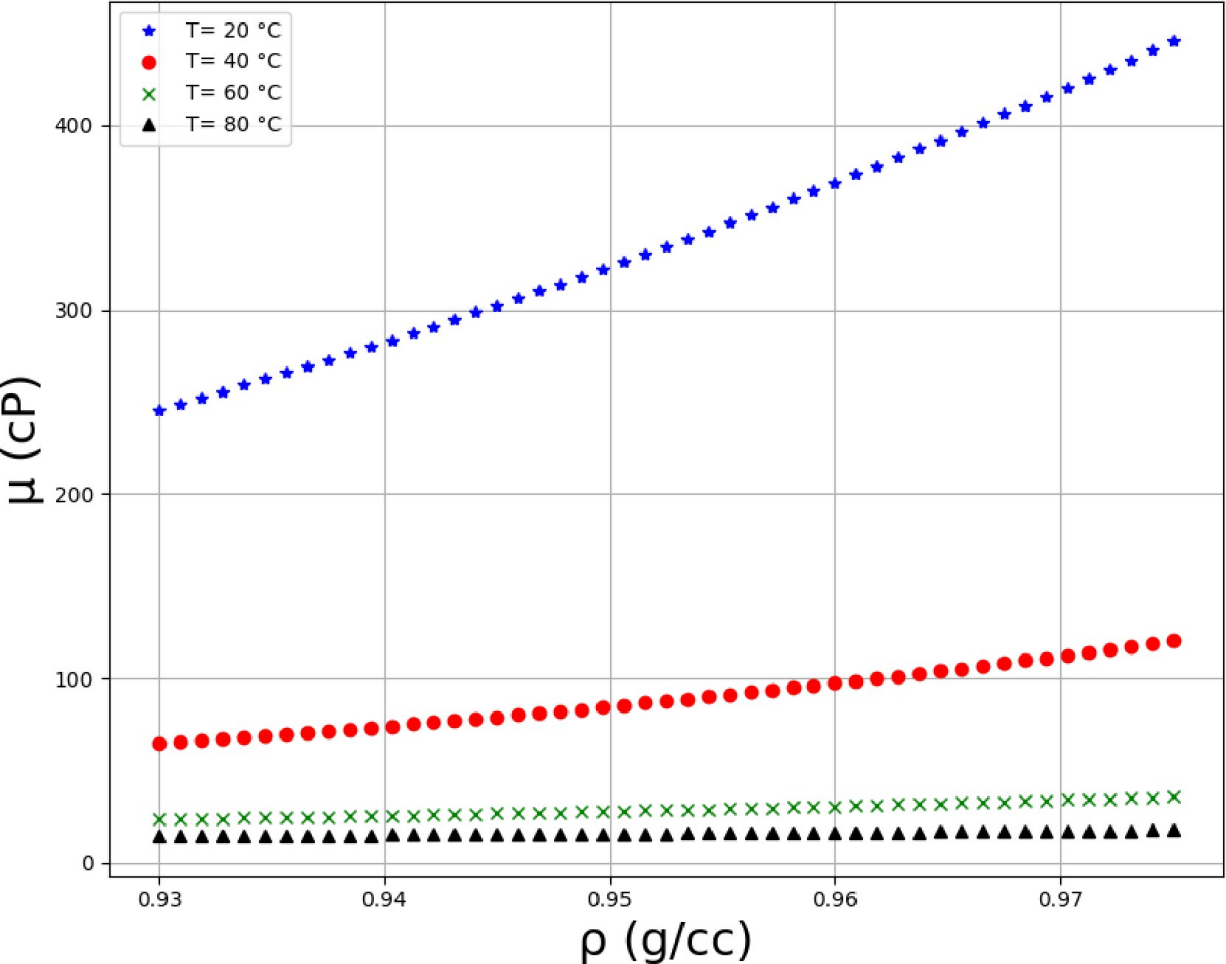
Trends of density on multiple temperature levels (API = 18.6, %C4 = 0.35, %C5 = 1.62, %C6 = 3.21, %C7+ = 94.81, SGC7+ = 0.95.

Using the MLP model, which is tuned using FA algorithm, the viscosity analysis was performed the results of which are illustrated in Figs 8–13 in form of 3D and 2D plots. As seen, the temperature has the most significant effect on the variations of crude oil viscosity and the value of viscosity is highly dependent on the temperature. Moreover, density and API of oils have significant effects on the viscosity after the temperature factor. It was also revealed that the variations of viscosity with the molecular weight and the oil compositions are not substantial, compared to other parameters. The results are in agreement with the previously reported correlations for the viscosity estimation using compositional data [2].

## 5. Conclusion

In the field of petroleum science, viscosity measurement of heavy crude oil is crucial, and reservoir simulators are commonly used for this purpose. In this study, multiple distinct models are used to predict the viscosity of heavy oil using the available data. The Decision Tree (DT), MLP, and GRNN models are used in this study, and the firefly algorithm (FA) is used to optimize the hyper-parameters of these models. For the final models of DT, MLP, and GRNN, the RMSE error rates are 40.52, 25.08, and 30.83, respectively. In addition, the respective R^2^-scores are 0.921, 0.978, and 0.933. MLP was selected as the best model for this study in estimating the oil viscosity via compositional data. Compared with research reported in [2], the result obtained from MLP is almost equal to the R^2^ criterion in the test, but it shows a better result in terms of other values in the test phase. This fact shows the effect of optimizing hyper-parameters and removing outliers on obtaining a better and more general model.
